# Fission Yeast Polarization: Modeling Cdc42 Oscillations, Symmetry Breaking, and Zones of Activation and Inhibition

**DOI:** 10.3390/cells9081769

**Published:** 2020-07-24

**Authors:** Bita Khalili, Hailey D. Lovelace, David M. Rutkowski, Danielle Holz, Dimitrios Vavylonis

**Affiliations:** 1 Department of Physics, Lehigh University, Bethlehem, PA 18015, USA; bitakhalili@protonmail.com (B.K.); hlovela@g.clemson.edu (H.D.L.); dmr518@lehigh.edu (D.M.R.); dah414@lehigh.edu (D.H.); 2 Department of Physics and Astronomy, Clemson University, Clemson, SC 29631, USA

**Keywords:** cell polarization, mathematical model, fission yeast, reaction–diffusion model, small GTPases, Cdc42 oscillations

## Abstract

Cells polarize for growth, motion, or mating through regulation of membrane-bound small GTPases between active GTP-bound and inactive GDP-bound forms. Activators (GEFs, GTP exchange factors) and inhibitors (GAPs, GTPase activating proteins) provide positive and negative feedbacks. We show that a reaction–diffusion model on a curved surface accounts for key features of polarization of model organism fission yeast. The model implements Cdc42 membrane diffusion using measured values for diffusion coefficients and dissociation rates and assumes a limiting GEF pool (proteins Gef1 and Scd1), as in prior models for budding yeast. The model includes two types of GAPs, one representing tip-localized GAPs, such as Rga3; and one representing side-localized GAPs, such as Rga4 and Rga6, that we assume switch between fast and slow diffusing states. After adjustment of unknown rate constants, the model reproduces active Cdc42 zones at cell tips and the pattern of GEF and GAP localization at cell tips and sides. The model reproduces observed tip-to-tip oscillations with periods of the order of several minutes, as well as asymmetric to symmetric oscillations transitions (corresponding to NETO “new end take off”), assuming the limiting GEF amount increases with cell size.

## 1. Introduction

The ability of cells to establish an axis for directed growth, motion, or mating relies on their ability to localize signaling proteins at the growing or leading edge of the cell. Such processes enable motile cells to migrate, epithelial cells to develop and maintain tissues, and neurons to grow axons and dendrites [1,2,3]. Cell polarization generally arises from symmetry-breaking formation of robust protein localization patterns along the cell membrane [4,5,6]. Small GTPases, such as Ras and Cdc42, play a central role in cell polarization by switching between active GTP-bound and inactive GDP-bound forms. A system of activators (GEFs) and inhibitors (GAPs) provide positive and negative feedbacks for small GTPase activation and inactivation [7,8,9,10]. Through self-organization, this results in the formation of membrane regions enriched in activated signaling proteins including Cdc42-GTP.

Extensive experimental and modeling studies in budding yeast, *S. cerevisiae*, have highlighted important mechanisms in polarization, namely formation of a stable patch along the cell membrane, the site of bud growth. These mechanisms are related to the process of Turing pattern formation [5,11,12,13]. Activated Cdc42 accumulates at a dominant patch where it forms slowly-diffusing aggregates (corresponding to a slowly diffusing local activation) that recruit the Cdc42 GEF, while at the same time depleting it from elsewhere in the cell (a type of global inhibition) [11]. The positive feedback through the “winner-take-all” mechanism is also enhanced by the actin system [14,15,16,17,18].

While polarization in budding yeast involves selection of a single growth site, some organisms can maintain multiple active sites. This includes rod-shaped fission yeast, *S. pombe*, a model organism for studies of cell shape. Many mutations perturb its normal tubular shape towards thinner, wider, round, T-shaped, banana-shaped or other shapes [19]. Studies of fission yeast cell polarity have highlighted several additional phenomena suggestive of a modeling approach:

(i) Fission yeast is able to maintain two stable sites of growth and Cdc42-GTP localization (at the two tips) rather than one. After cell division, a fission yeast cell begins monopolar growth from the old end inherited from the mother cell. The cell subsequently experiences new end take off (NETO) and enters a bipolar growth phase from both cell tips [20]. NETO transition has been described as a result of competition over a limiting component between the two tips that can reach saturation [21,22,23,24].

(ii) Cdc42 oscillatory and fluctuating states underlie the monopolar and bipolar growth states of fission yeast [22,25] (reminiscent of the Min protein oscillation system in bacteria [26,27]). During mating, Ras1/Cdc42 patch appearance and disappearance dynamics are also crucial for cells to find and polarize towards a mating partner [28]. These observations suggest that Cdc42 oscillations and fluctuations embody an exploratory mechanism, enabling cells to adapt their growth pattern in response to external and internal cues to maximize cell survival [22,29].

(iii) The two Cdc42 GEFs, Scd1 and Gef1, localize at cell tips, together with Cdc42-GTP [22,30,31,32,33,34,35]. By contrast, three known Cdc42 GAPs establish an intriguing pattern, with Rga4 [36,37,38] and Rga6 [39] decorating primarily the cell sides while Rga3 accumulates primarily at the cell tips [40]. Fluorescence recovery after photobleaching (FRAP) studies indicate different dynamics of Rga6 at cell tips as compared to cell sides: the percent recovery at the cell sides is smaller than the tips over the same time period [39].

(iv) Recent evidence suggests that localization of Cdc42-GTP and Ras1-GTP to cell tips occurs primarily through fast membrane diffusion of Cdc42-GDP and Ras1-GDP, converting to slowly-diffusing GTP forms at the cell tips [9,33,41]. While polarization is generally thought to require Cdc42-GDP extraction from the cell membrane through guanine nucleotide dissociation inhibitor (GDI), the effect of the fission yeast GDI Rdi1 is relatively small as cells are able to polarize in its absence [33]. Membrane diffusion coefficients and dissociation rates of Cdc42 have been previously estimated and can be incorporated into models [33,41].

In this short article, we focus on the broad dynamic and geometric features of the fission yeast polarization system to propose a reaction–diffusion model that can account for the polarity transitions and spatial pattern of Cdc42, its activators and inhibitors. The aim of this top-down approach is to (i) indicate the minimum level of complexity required to describe the broad features mentioned above, (ii) motivate experiments to measure unknown model parameters, and (iii) serve as a framework to more accurately incorporate missing biological mechanisms. Compared to previous models of fission yeast polarization [21,22,23,24,42,43,44,45], here we include GAP localization on the cell membrane, we use diffusion coefficients of Cdc42-GTP and Cdc42-GDP on the cell membrane estimated in experiments, and we implement the 3D geometry of the rod-shaped fission yeast.

To model GAP plasma membrane recruitment, we suggest a mechanism similar to our previous model of localized membrane recruitment of Gap1, the GAP of Ras1, at the exploratory Ras1 patch during fission yeast mating [41]. The effect of local Gap1 recruitment and diffusion around the mating patch was to restrict the zone of Ras1 activation and regulate the lifetime of the exploratory patch [41,46].

## 2. Model

**Figure 1:**
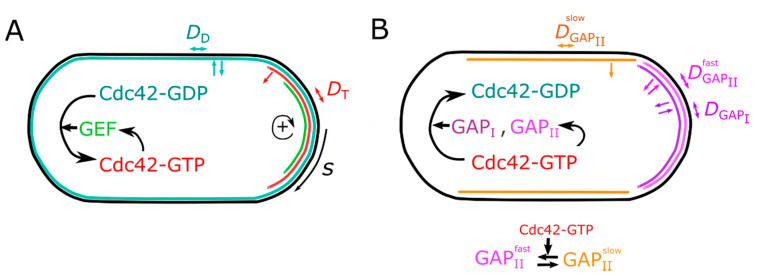
Modeling Cdc42 activation and regulator distribution. (**A**) Schematic illustrates GEF-mediated positive feedback at cell tips, with colored regions indicating zones of active Cdc42-GTP patch. The model equations are identical for both tips; however, they allow symmetry-breaking states and one dominant tip as shown. Parameter *s* indicates arc length distance from the nearest cell tip. Colored arrows indicate association, dissociation, and diffusion along cell membrane. Cdc42-GDP (teal) associates, dissociates, and diffuses on the plasma membrane with diffusion coefficient $D_{D}$. It converts to slowly-diffusing Cdc42-GTP (red, diffusion coefficient $D_{T}$) by GEF (green) that is recruited to the membrane by Cdc42-GTP in a nonlinear manner, establishing a positive feedback (+ arrow). (**B**) Schematic illustrates the negative feedback through GAPs. GAP_I_ (purple, diffusion coefficient $D_{{\mathrm{GAP}}_{I}}$) and fast-diffusing GAP_II_ (pink, diffusion coefficient $D_{{\mathrm{GAP}}_{\mathrm{II}}}^{\mathrm{fast}}$) are recruited to the membrane through Cdc42-GTP. Fast-diffusing GAP_II_ spontaneously converts to slow-diffusing GAP_II_ (orange, diffusion coefficient $D_{{\mathrm{GAP}}_{\mathrm{II}}}^{\mathrm{slow}}$), while the reverse (slow to fast) is catalyzed by Cdc42-GTP. All GAPs catalyze hydrolysis of Cdc42-GTP.

We developed a system of partial differential equations that implements diffusion on a 3D curved surface representing a fission yeast plasma membrane. We used the same numerical methods as an earlier study for reaction–diffusion of Ras1 during cell mating [41,47]. In the simulations we compute the surface concentrations of Cdc42-GDP, $\;C_{D}$, and Cdc42-GTP, $C_{T}$ through diffusion and reaction (Figure 1). The surface representing the plasma membrane has the shape of fission yeast cells with a cylindrical body of radius 2 μm capped by hemispherical tips at either end. We do not implement a reaction scheme for Ras1, so in this preliminary model $\;C_{T}$ and $C_{D}$ can be thought to represent the combined Ras1/Cdc42 polarity patch. The activation and deactivation of Cdc42 is regulated by the surface concentrations of GEFs, *C*_GEF_, and two types of GAPs: GAP_I_, $C_{{\mathrm{GAP}}_{I}},$ and GAP_II_ that we assume exists in two distinct diffusive states, $C_{{\mathrm{GAP}}_{\mathrm{II}}}^{\mathrm{fast}}$ and $C_{{\mathrm{GAP}}_{\mathrm{II}}}^{\mathrm{slow}}$, to be described below. The equations describing Cdc42 dynamics are as follows:

(1) $$\partial C_{D}/\partial t=D_{D}{∆}_{S}C_{D}+j_{D}^{p}+(k_{1}^{n}+k_{2}^{n}C_{{\mathrm{GAP}}_{I}}+k_{3}^{n}C_{{\mathrm{GAP}}_{\mathrm{II}}}^{\mathrm{fast}}+k_{8}^{n}C_{{\mathrm{GAP}}_{\mathrm{II}}}^{\mathrm{slow}})C_{T}-k_{0}^{p}e^{-\frac{s}{\lambda }}C_{\mathrm{GEF}}C_{D}\;-r_{D}C_{D}-r_{\mathrm{noise}}C_{D},$$

(2) $$\partial C_{T}/\partial t=D_{T}{∆}_{S}C_{T}+k_{0}^{p}e^{-\frac{s}{\lambda }}C_{\mathrm{GEF}}C_{D}-(k_{1}^{n}+k_{2}^{n}C_{{\mathrm{GAP}}_{I}}+k_{3}^{n}C_{{\mathrm{GAP}}_{\mathrm{II}}}^{\mathrm{fast}}+k_{8}^{n}C_{{\mathrm{GAP}}_{\mathrm{II}}}^{\mathrm{slow}})C_{T}-r_{T}C_{T}+r_{noise}C_{D}.$$

Here ${∆}_{S}\;$ is the Laplace–Beltrami operator, and *D* here and below represents diffusion coefficients. Symbols *k* and *r* indicate reaction and membrane dissociation rate constants, respectively. Superscripts *p* and *n* indicate positive and negative feedback contributions, respectively. Constant rate $j_{D}^{p}$ represents uniform association of Cdc42-GDP to the plasma membrane from a cytoplasmic pool, which we assume has a constant concentration. We used prior experimentally estimated diffusion coefficients and membrane dissociation constants $r_{D}$ and $r_{T}$, which are assumed to implicitly include the effect of GDI Rdi1. In Equations (1) and (2), GAPs promote conversion of Cdc42-GTP to Cdc42-GDP and the reverse conversion is promoted by GEFs. Activation of Cdc42 by GEFs is biased to occur close to the cell tips, where it should be enhanced through microtubule-based tip delivery of Tea proteins, restriction of activation zone through the endoplasmic reticulum (ER), and possibly actin polymerization [32,48,49,50]. This is implemented through the exponential term where *s* is the arc length distance to the closest tip (Figure 1) and parameter λ indicates the scale over which activation at cell tips is assumed to occur. The model allows for random activation of Cdc42-GDP with rate $r_{noise}$, implemented similarly as in [41].

Accumulation of GEFs in response to Cdc42-GTP is assumed to occur through an autocatalytic mechanism that has a functional form similar to the positive feedback proposed for *S*. *cerevisiae* polarization [11]. In this positive feedback mechanism, a finite amount of GEF in the system is assumed to be distributed in quasi-static equilibrium with higher proportions at sites with higher active Cdc42 concentration:

(3) $$C_{\mathrm{GEF}}=k_{1}^{p}E_{c}C_{T}/V+k_{2}^{p}E_{c}C_{T}^{2}/V,$$

(4) $$E_{c}=E_{c}{}^{\mathrm{tot}}/(1+\int \left[k_{1}^{p}C_{T}/V+k_{2}^{p}C_{T}^{2}/V\right]da),$$

where $E_{c}$ is the available number of GEF molecules in the cytoplasm, $E_{c}{}^{\mathrm{tot}}$ is the total number of GEF molecules in the cell, and *V* is the cell volume. In Equation (4), the integral is over the cell’s surface area. The quasi-static approximation is introduced for simplicity, to avoid additional parameters related to a GEF concentration field in the model; earlier work has shown this approximation is valid in the limit of sufficiently fast GEF membrane dissociation rate [11,41]. Membrane-bound Cdc42 GEFs Scd1 and Gef1 are indeed localized at the cell tip, where they are expected to form complexes with Cdc42-GTP [22,30,31,35]. Within the simplifying quasi-static approximation, we are thus consistently assuming a GEF membrane diffusion coefficient similar to that of Cdc42-GTP. The nonlinear dependence in Equation (4) leads to a positive feedback strong enough to break symmetry and establish a Cdc42-GTP patch [5], and is supported by experiments showing recruitment of GEF Scd1 depends on scaffold protein Scd2, which itself depends on Cdc42-GTP [35].

In the model, we include a negative inhibitor that we designate GAP_I_, which accumulates at cell tips through Cdc42-GTP-mediated recruitment, and provides a nonlinear negative feedback able to generate fluctuations and oscillations. Including such a component is motivated by the observed tip localization of Rga3 [40], but we also bundle together all tip-localized inhibition mechanisms of Ras1 and Cdc42 through Gap1, Pak1 and actin. We assumed a functional form similar to the Ras1 GAP, Gap1, recruitment to the exploratory mating patch [41] and the negative feedback for Cdc42 oscillations in budding yeast [51]:

(5) $$\partial C_{{\mathrm{GAP}}_{I}}/\partial t=D_{{\mathrm{GAP}}_{I}}{∆}_{S}C_{{\mathrm{GAP}}_{I}}+k_{4}^{n}C_{T}^{h}/(k_{\mathrm{sat}}^{h}+C_{T}^{h})-r_{{\mathrm{GAP}}_{I}}C_{{\mathrm{GAP}}_{I}}\;.$$

The second term on the right hand side represents cooperative recruitment at small Cdc42-GTP concentrations, reaching a plateau for concentration above $k_{sat}.\;$ We used a value *h* = 2 that was sufficient to provide delayed negative feedback needed for oscillations.

To generate a spatial pattern of inhibitors such as Rga4 and Rga6, which accumulate in “collar” or “corset” shapes around growing cell tips [36,37,38,39], we make the bold assumption that these inhibitors that we collectively call GAP_II_ are also recruited to the plasma membrane through Cdc42-GTP (similar to GAP_I_). We further assume that GAP_II_ proteins that diffuse away from the cell tip convert to slowly-diffusing forms, possibly through binding to each other, thus accumulating away from the active region. Support for such a differential mobility along the plasma membrane is the observation of larger FRAP recovery of Rga6 at cell tips compared to cells sides, and a pattern of FRAP recovery consistent with Rga6 membrane diffusion [39]. The dynamics of the fast and slow GAP_II_ components are described by:

(6) $$\partial C_{{\mathrm{GAP}}_{\mathrm{II}}}^{\mathrm{fast}}/\partial t=D_{{\mathrm{GAP}}_{\mathrm{II}}}^{\mathrm{fast}}{∆}_{S}C_{{\mathrm{GAP}}_{\mathrm{II}}}^{\mathrm{fast}}+k_{5}^{n}C_{T}-k_{6}^{n}C_{{\mathrm{GAP}}_{\mathrm{II}}}^{\mathrm{fast}}+k_{7}^{n}C_{{\mathrm{GAP}}_{\mathrm{II}}}^{\mathrm{slow}}C_{T}-r_{{\mathrm{GAP}}_{\mathrm{II}}}^{\mathrm{fast}}C_{{\mathrm{GAP}}_{\mathrm{II}}}^{\mathrm{fast}}\;,$$

(7) $$\partial C_{{\mathrm{GAP}}_{\mathrm{II}}}^{\mathrm{slow}}/\partial t=D_{{\mathrm{GAP}}_{\mathrm{II}}}^{\mathrm{slow}}{∆}_{S}C_{{\mathrm{GAP}}_{\mathrm{II}}}^{\mathrm{slow}}+k_{6}^{n}C_{{\mathrm{GAP}}_{\mathrm{II}}}^{\mathrm{fast}}-k_{7}^{n}C_{{\mathrm{GAP}}_{\mathrm{II}}}^{\mathrm{slow}}C_{T}-r_{{\mathrm{GAP}}_{\mathrm{II}}}^{\mathrm{slow}}C_{{\mathrm{GAP}}_{\mathrm{II}}}^{\mathrm{slow}}.$$

Both fast and slow forms can hydrolyze Cdc42-GTP, at different rates as shown in Equations (1) and (2). We also assumed that Cdc42-GTP can catalyze conversion of $C_{{\mathrm{GAP}}_{\mathrm{II}}}^{\mathrm{slow}}$ and $C_{{\mathrm{GAP}}_{\mathrm{II}}}^{\mathrm{fast}}$ through the $k_{7}^{n}$ terms in Equations (6) and (7) (since otherwise the distribution of $C_{{\mathrm{GAP}}_{\mathrm{II}}}^{\mathrm{slow}}$ would be peaked at the tips instead of away from cell tips). Though we are not aware of experimental evidence in support of the latter assumption, this process might occur through release of slow $C_{{\mathrm{GAP}}_{\mathrm{II}}}^{\mathrm{slow}}$ from a protein complex after binding to Cdc42-GTP.

For most of this study we kept cells at a fixed reference length of 8 μm, and other parameters as in Table 1. The area of each Voronoi cell used in the discretization of the surface area was between 0.017 to 0.046 μm^2^. We used a simulation time step of 0.01 s and started the simulations from an unpolarized state, with Cdc42-GDP at the concentration it would have at steady state in the absence of activation ($C_{D}=j_{D}^{p}/r_{D})$ plus or minus small relative random fluctuations. We also initialize a smaller random $C_{T}\;$ field and checked the evolution of the system over hundreds or thousands of seconds.

## 3. Results

### 3.1. Goals

We explored our model’s ability to capture basic phenomenology of fission yeast Cdc42 polarization by adjusting the unknown rate constants while using reported estimates for the membrane diffusion coefficients and dissociation rates of Cdc42-GTP and Cdc42-GDP. The desired phenomena include: (i) ability of the system to exhibit asymmetric, symmetric, and oscillatory states; (ii) a pattern of polarity transitions as function of changing rate constants matching fission yeast polarity change with cell growth; (iii) oscillatory states with periods in the range of 4–6 min [22]; (iv) enhancement of Cdc42 concentration (combined Cdc42-GDP and Cdc42-GTP) by 2–3-fold at cell tips compared to cell sides [33]; (v) establishment of micron-scale active regions at cell tips [22,32,33,52]; (vi) accumulation of GAP_I_ at cell tips and GAP_II_ in collar/corset manner away from cell tips.

### 3.2. Dynamical States Observed in Parameter Scan

Through a systematic but non-exhaustive scan of unknown rate constants (see Table 1), we were able to show that the system of Equations (1)–(7) provides a mechanism with solutions that can describe, to different extents, all the desired phenomena mentioned above. The model also accounted for the geometric features of fission yeast cells through our implementation of reaction–diffusion equations on a curved surface.

**Figure 2:**
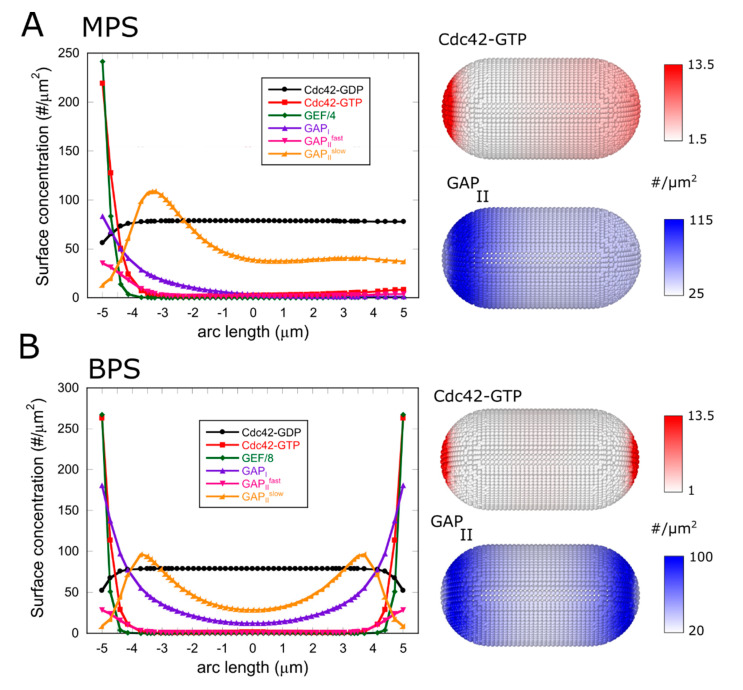
Examples of asymmetric stationary (monopolar stable, MPS) and symmetric stationary (bipolar stable, BPS) solutions. The system evolved to these stationary states over a time course in the order of hundreds of seconds from an initially unpolarized state. (**A**) Concentrations as a function of arc length distance from cell tip and snapshots showing the concentration of Cdc42-GTP and GAP_II_ (sum of $C_{{\mathrm{GAP}}_{\mathrm{II}}}^{\mathrm{slow}}$ and $C_{{\mathrm{GAP}}_{\mathrm{II}}}^{\mathrm{fast}}$). Parameter values as in Table 1, except for $k_{\mathrm{sat}}$ = 900/μm^2^ and $E_{C}^{\mathrm{tot}}=700.$ (**B**) Same as panel A but for a BPS state. Parameter values same as in Table 1, except for $E_{C}^{\mathrm{tot}}=1800$.

For a large range of model parameter values, the system converged to stationary solutions that were either asymmetric (MPS, monopolar stable) or symmetric (BPS, bipolar stable), as shown in Figure 2. Because both tips have identical rate constants, the MPS states represent symmetry-breaking states. The zones of activation were always found at one or both of the tips since we bias Cdc42 activation to the cell tip region. 

For the MPS and BPS examples in Figure 2, the concentration of Cdc42 at activated cell tips is enhanced by a factor of 2–3 compared to cell sides: This is due to accumulation of Cdc42-GTP at cells tips, consistent with prior experiments [33]. The concentration profile of Cdc42-GDP exhibits a small dip closer to the active cell tip: This reflects the diffusive flux of Cdc42-GDP towards the active cell tip that is balanced by diffusive flux and dissociation of Cdc42-GTP away from the tip. In the MPS states, the lagging tip maintained active Cdc42 at lower concentrations compared to the dominant tip.

The profiles of Figure 2 also show the localization of GAP_I_ at the active cell tip and GAP_II_ in a collar/corset manner away from the cell tips. The concentration of GAP_II_ peaks around the active tip region, which is in qualitative agreement with profiles of Rga4 in microscopy images [36,38] (though perhaps more exaggerated compared to experiments).

**Figure 3:**
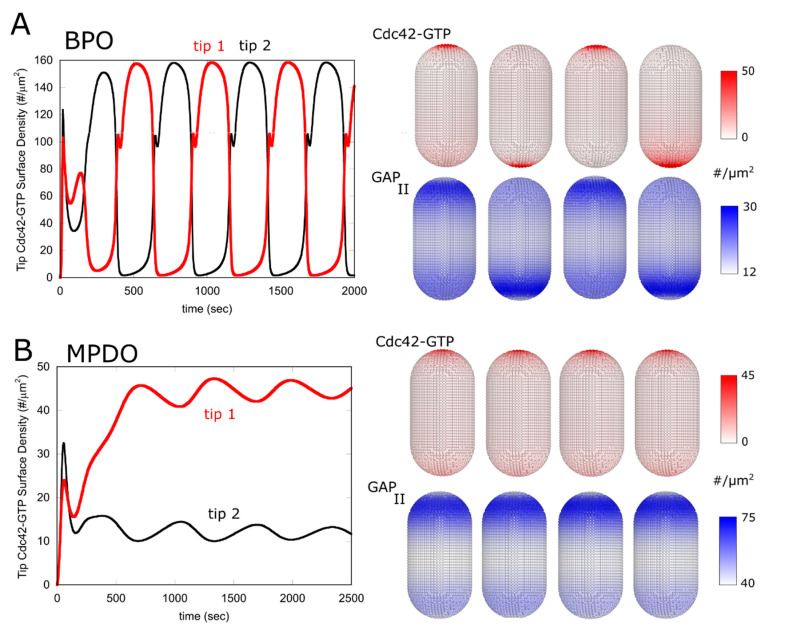
Examples of symmetric oscillations (bipolar oscillatory, BPO) and asymmetric damped oscillations (monopolar with damped oscillations, MPDO) solutions. (**A**) Graph shows concentration of Cdc42-GTP at each tip, as a function of time starting from an unpolarized state with both tips inactive. The concentration profiles of the other components in the system that are not shown follow the same trends as in the stationary states of Figure 2. Snapshots show Cdc42-GTP and total GAP_II_ profiles every 200 s once the system evolved to a periodic pattern. Parameter values same as shown in Table 1 except $k_{5}^{n}$ = 0.01/s and $E_{C}^{\mathrm{tot}}=500.$ (**B**) Same as panel A, but for an MPDO case where the system eventually evolves to a stationary asymmetric state through damped, anticorrelated oscillations. Snapshots show Cdc42-GTP and total GAP_II_ profiles every 200 s, starting at 1000 s. Parameter values same as shown in Table 1, except $E_{C}^{\mathrm{tot}}=210$.

Another common solution of the system was states exhibiting symmetric anticorrelated oscillations, as shown in Figure 3A and Video S1. These BPO (bipolar oscillatory) states had similar active tip concentration profiles to the stationary states of Figure 2; however, the dominant active tip kept switching from one tip to the other, as observed during Cdc42 fluctuations and oscillations after NETO [22].

A characteristic feature of Cdc42 dynamics in fission yeast is the anticorrelated fluctuations and oscillations of Cdc42-GTP before NETO, a period of the cell cycle where cells grow in a monopolar manner and Cdc42 activity is larger at the old end [22]. We found that such dynamical states are also observed by our proposed dynamics, though a finer tuning of parameters is required as compared to MPS, BPS and BPO states. We found that the system of Equations (1)–(7) can generate asymmetric oscillations that persist undamped; however, within our desired set of criteria listed at the beginning of the Results section, we only found asymmetric damped oscillatory states (MPDO), as shown in the example of Figure 3B. Additional sources of noise in cells (not included in our model) may convert those states to long lived anti-correlated fluctuations and oscillations [43,53]; thus we interpret the existence of MPDO dynamics as consistent with the asymmetric oscillations and fluctuations of Cdc42-GTP before NETO.

### 3.3. Structure of Solutions in Parameter Space

**Figure 4:**
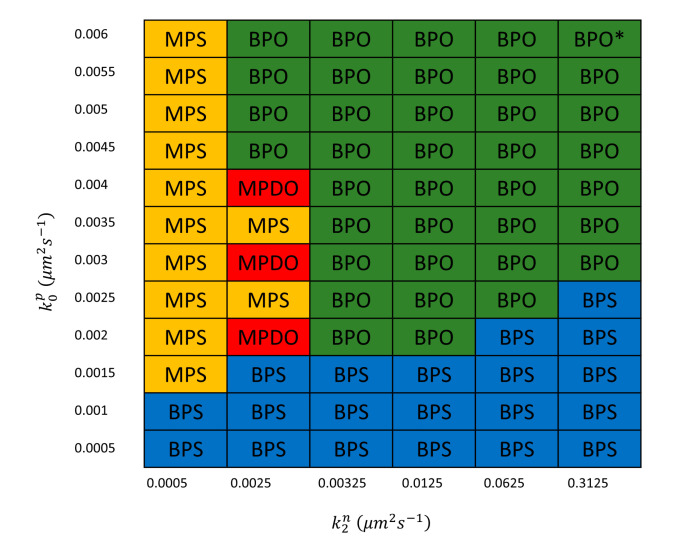
Structure of dynamical states reached, as function of a positive ($k_{0}^{p}$) and negative ($k_{2}^{n}$) feedback rate constants. Results show the final state reached by a single simulation for each set of parameters over time course of 800–1200 s, starting from an unpolarized state. In state marked with *, the Cdc42-GTP patch regions becomes as small as a single Voronoi region of the discretized cell surface. Other parameters same as Table 1.

Since fission yeast cells transition from asymmetric Cdc42 oscillations and fluctuations prior to NETO to symmetric oscillations and fluctuations after NETO [22], the MPDO and BPO states are biologically relevant. To better understand the requirements to observe such states in the simulations, we varied two parameters, $k_{0}^{p}$ and $k_{2}^{n}$, describing the strength of positive and negative feedbacks in the system, focusing on a region around the rarer MPDO solutions, as shown in Figure 4. We found that all of the dynamical states of Figure 2 and Figure 3 arose in the neighborhood of MPDO, with higher values of both $k_{0}^{p}$ and $k_{2}^{n}$ resulting in BPO. MPDO states were observed in between MPS and BPO states when the examined positive feedback parameter was above a threshold; below the threshold, the system results in weakly active BPS states. We note that along the boundaries of dynamical state regions of Figure 4, the system might settle to a different pattern from run to run, which indicates regions with multiple solutions may “co-exist” [22]. We did not explore the coexistence behavior in detail in this work.

**Figure 5:**
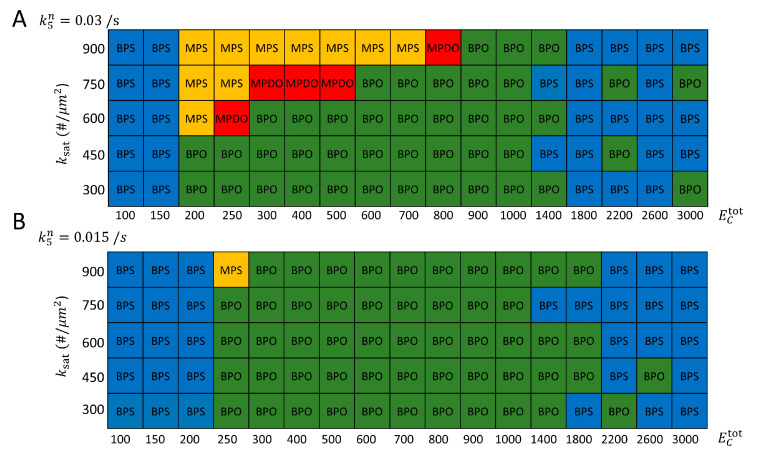
Structure of dynamical states reached, as function of total GEF amount ($E_{C}^{\mathrm{tot}}$) and negative feedback parameter $k_{\mathrm{sat}}$. Results show the final state reached by a single simulation for each set of parameters over time course of 800–1200 s, starting from an unpolarized state. Other parameters same as Table 1. (**A**) Reference simulations. For $k_{\mathrm{sat}}$ = 600−900/μm^2^, the system can transition from MPDO to BPO with increasing $E_{C}^{\mathrm{tot}}$, a behavior consistent with the dynamical change observed under cell growth around NETO. (**B**) Same as panel A but reducing the rate of GAP_II_ recruitment to the cell tips, as might occur in *rga4Δ* or *rga6Δ* cells. The BPO region is observed to expand compared to panel A, while the region corresponding to MPS and MPDO states shrinks.

We next asked if the model is consistent with transition from MPDO to BPO around NETO. We anticipate the MPDO to BPO transition to occur as a result of the increase of a limiting component with cell growth. We thus, varied the parameter that describes the total amount of GEF in the system,$\;E_{C}^{\mathrm{tot}}$, together with a parameter that influences the negative feedback strength, $k_{\mathrm{sat}}$, to help unfold the pattern of dynamical transitions in Figure 5A. We observed that, indeed, for $k_{\mathrm{sat}}$ above 600/μm^2^ there is a large region in parameter space corresponding to MPDO, shifting to BPO as the total amount of GEF in the system is doubled. Interestingly, at even larger $E_{C}^{\mathrm{tot}}$ (above 1400) the system reverts to BPS. This is consistent with the uncorrelated small fluctuations of active Cdc42 in long *cdc25-22* cells [22].

**Figure 6:**
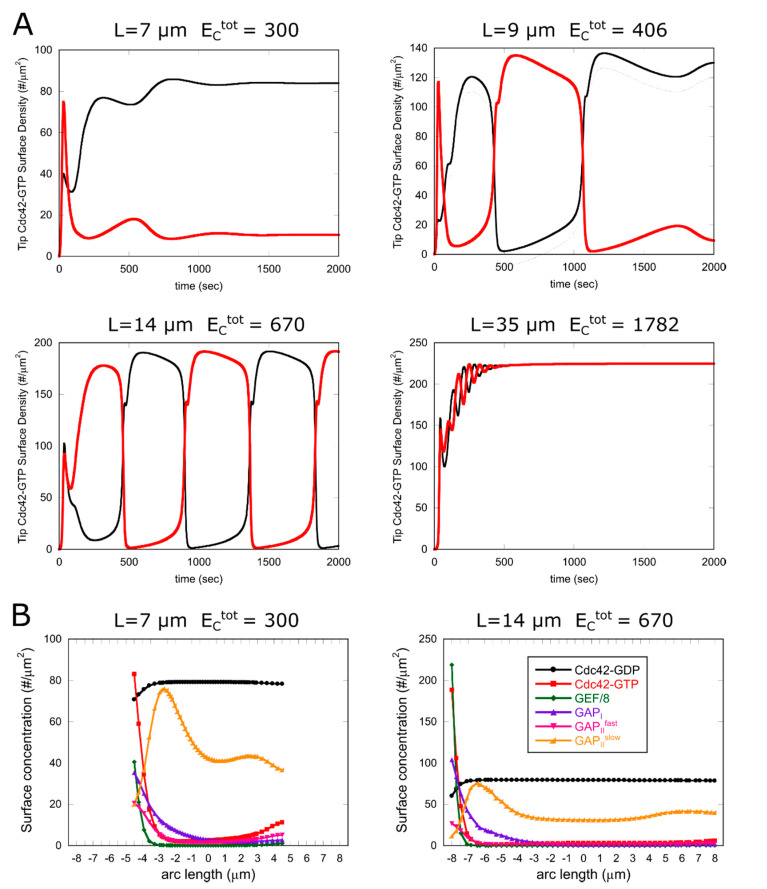
Results of simulations at different fixed cell lengths, *L,* and increasing the total GEF in proportion to cell volume, as indicated in each graph. Other parameters same as Table 1, except for $k_{\mathrm{sat}}$ = 650/μm^2^. (**A**) Plots of Cdc42-GTP concentration at each tip (red and black curve) as a function of time starting from an unpolarized state with both tips inactive. System transitions from MPDO to BPO and BPS with increasing *L*. (**B**) Concentrations as a function of arc length distance from cell tip for a cell of length 7 μm (left, at steady asymmetric state) and length 14 μm (right, after 1060 s of simulation of cell undergoing symmetric oscillations, at an instant with a dominant left tip).

To further demonstrate how the model captures the transition around NETO, we used the parameter values suggested from Figure 5A, to perform simulations at different fixed cell lengths with $k_{\mathrm{sat}}$ = 650/μm^2^ and increasing the total GEF in proportion to cell volume such that $E_{C}^{\mathrm{tot}}=300\;$ for cell length *L* = 7 μm. Indeed, the simulations of Figure 6A show a transition from MPDO to BPO at around the cell length where NETO occurs (9.5 μm [20]). Very long cells with *L* = 35 μm in Figure 6A revert to BPS, consistent with the behavior of long *cdc25-22* cells [22], as mentioned in the preceding paragraph. The distribution of Cdc42, GEF and GAPs maintains the same features with increasing length, with the Cdc42 patch becoming more intense and slightly narrower with increasing length (Figure 6B).

### 3.4. Simulations with Reduced GAP_II_ Recruitment

Motivated by prior experimental studies of Rga4 and Rga6 deletion mutants [22,36,39], in our model we reduced the rate of GAP_II_ recruitment to the cell tips by a factor of two (described by parameter $k_{5}^{n})$. We repeated the scan of the values of $E_{C}^{\mathrm{tot}}$ and $k_{\mathrm{sat}}$ and plot the resulting states in Figure 5B. Interestingly, this change lead to an expansion of the BPO region, a significant shrinkage of the MPS region and disappearance of MPDO states (within the resolution of the scan).

The behavior observed in Figure 5, when reducing parameter $k_{5}^{n}$, does not appear to closely relate to prior experimental observations: *rga4Δ* cells demonstrate more pronounced symmetric oscillations compared to wild-type; however, they also exhibit asymmetric states [22,36]. Meanwhile *rga6Δ* cells have been found to be slightly more monopolar, with smaller relative Cdc42 fluctuations compared to wild-type [39]. This comparison to experiments suggests that there is more to polarity regulation of these mutant cells than simply changing a single rate constant of our model (see recent results in [54]).

**Figure 7:**
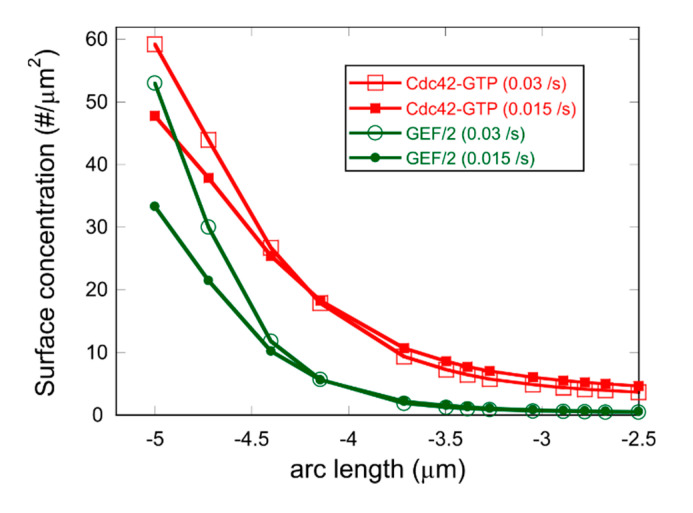
Plot of Cdc42-GTP and GEF concentration gradient when the rate of GAP_II_ recruitment to the cell tips is $\;k_{5}^{n}=0.03\;/s$ (empty symbols) and for the same parameters with reduced rate of $k_{5}^{n}=0.015\;/s\;$ (solid symbols), as might occur in *rga4Δ* cells. Other parameters are the same as Table 1 except $k_{\mathrm{sat}}=450\;/{\mu m}^{2}$, such cases result in BPO oscillations (see Figure 5B). This plot shows the concentration profiles at the dominant tip ~ 20 s after Cdc42-GTP tip concentration peaks, and after at least 1000 s of simulation time. The half-widths are larger with reduced GAP_II_ recruitment rate: An exponential fit to the Cdc42-GTP profile gives a decay length 0.64±0.01 μm and 0.72±0.01 μm, respectively. The left-most point on the x axis corresponds to the left tip, as in Figure 2.

The model, nevertheless, captures some of the properties of *rga4Δ* and *rga6Δ* cells when plotting the Cdc42-GTP and GEF profiles around an active tip (Figure 7). We find a wider profile of Cdc42 activation, as observed in *rga4Δ* cells that have larger cell diameter compared to wild-type cells [32,52]. Similarly, *rga6Δ* have been found to be wider than wild-type cells and *rga4*Δ*rga6*Δ double mutants are wider than either single mutants [39].

## 4. Discussion

The model presented in this work generalizes the delayed differential equations (DDE) model of Das et al. [22] for fission yeast. To reproduce observed Cdc42 dynamics, the previous model included competition between the two tips for a limiting component, assumed the existence of a positive activation feedback, assumed a maximum (or saturation) of tip activity, and included negative feedback through an explicit time delay. By assuming that the limiting component increases in amount with cell growth, this prior DDE model generated the transition from asymmetric to symmetric oscillations (identified here as MPDO and BPO states, respectively) that occurs with cell growth. The properties of such a DDE system together with diffusion along one dimension have been studied in detail by Xu and Bressloff [42]. Cerone et al. [23] also performed a detailed analysis of tip competition without oscillations at a level of ordinary differential equations.

In the current system of partial differential equations formulated on a surface in the shape of fission yeast, we associate the limiting component of Das et al. [22] with the GEF system, assuming a functional form for the positive feedback borrowed from studies of budding yeast polarization [11]. This limiting component enables transition from asymmetric to symmetric states with increasing amount of GEF. Unlike Das et al. we do not have an explicit parameter to saturate tip concentration and we do not have explicit time delay. These properties are assumed to be provided by the GAP system, for which we assume nonlinear dynamics similar to a prior model of transient Cdc42 patch competition in budding yeast [51]. Motivated by Khalili et al. [41], where we studied how Ras1 patch scans the cell membrane through appearance and disappearance, we assumed that GAPs of Cdc42 are recruited to the cell tip through Cdc42-GTP, similar to the recruitment of Gap1 to the mating patch, by Ras1-GTP [41,46].

Here we associated the nonlinear negative feedback necessary for emergence of oscillations with the function of tip-localized GAPs (“GAP_I_”). However we note that deletion of the tip-localized Rga3 did not significantly change Cdc42 oscillations compared to wild-type cells [40]. While it is also conceivable that negative regulation occurs by diffusion-limited supply of Cdc42-GDP at activated cell tips, this depletion is relatively small in Figure 2. In this figure, Cdc42-GTP accumulates at cell tips at concentrations that exceed Cdc42-GDP concentration at cell sides by a factor of 2–3, as observed experimentally [33] when using diffusion coefficients close to experimental estimates [33]. One important component that we did not include explicitly is Pak1 kinase, which has been proposed to mediate negative regulation through GEF phosphorylation in both fission and budding yeast [22,55,56], and, more recently, in positive feedback regulation in fission yeast [35].

We modeled accumulation of Rga4 and Rga6 along the cell sides by recruitment of a fast-diffusing GAP_II_ (representing both of these proteins) at cell tips, spontaneous conversion of fast-diffusing GAP_II_ to a slow-diffusing form, and conversion of slow- to fast-diffusing GAP_II_ in the activated tip region. While such a mechanism can lead to the desired effect of a collar of enhanced concentration of GAP_II_ around the growing tip, additional experimental support of such differential mobility is still needed. The postulated GAP_II_ mechanism may relate to how proteins Pom1 and Tea4, which peak at active cell tips, bind Rga4 and negatively regulate it away from active cell tips [48,50], and the actin-dependence of Rga6 recruitment [39], though we did not explicitly include them in the model. Direct binding of Rga4 and Rga6 to the cell sides from the cytoplasm could be an additional mechanism that should be incorporated into the model to better capture the polarity process.

**Figure 8:**
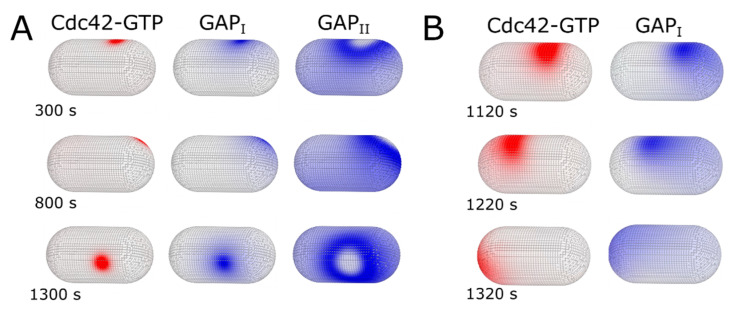
Snapshots of model results when Cdc42 activation is not biased at cell tips (limit of λ→∞). With other parameters same as Table 1, the system towards uniform activation. (**A**) Patch appearance and disappearance for $k_{0}^{p}$ = 0.01 μm^2^/s increased four times compared to Table 1. (**B**) Traveling wave behavior with parameters from Table 1 except $k_{0}^{p}$ = 0.01 μm^2^/s and $k_{5}^{n}=0$, which eliminates GAP_II_.

An important assumption in the model was that Cdc42 activation is biased towards cell tips. We can relax this assumption by taking the limit of parameter λ being very large. With the reference parameter values of Table 1, this results in uniform Cdc42 activation and loss of polarization. A further increase in positive feedback rate constant $k_{0}^{p}$ enables symmetry breaking: the model can then readily generate localized stable or oscillating patches of Cdc42-GTP, GEF, and GAP_I_, surrounded by a ring of high concentration of GAP_II_, forming at random locations on the simulated membrane (Figure 8A). This behavior of the model may relate to how loss of Orb6 kinase results in round cell morphologies by directing cell growth to the cell sides: Orb6 has been implicated in excluding Gef1 from accumulation to cell sides [31] and enhancing positive feedback at cell tips through Ras1 [57], in addition to other functions [58]. Stable side patch formation in the absence of cell tip bias may also relate to how T-shaped mutants initiate side projections [21].

The plots of Figure 2, Figure 3, Figure 4, Figure 5, Figure 6 and Figure 7 show estimated concentrations; however, we note that these numbers can be rescaled by adjustment of the unknown rate constants. In the model we accounted for whole-cell mass conservation of the limiting component, the GEF, but assumed a constant cytoplasmic concentration for everything else. Future improvements of the model would include accounting for mass conservation of all the system’s components, as well as for the free energy flow associated with GTP hydrolysis and non-equilibrium transport required to maintain concentration gradients. The model proposed here, together with previous modeling efforts, could also serve as a starting point for further quantitative investigations that include additional biological components that influence polarization, including the cytoskeleton [33], Gef1 phosphorylation [52], vesicle trafficking, and ER [49], as well as independent consideration of Ras1 from Cdc42 [35].

Our model implements similar mechanisms proposed for budding yeast polarization [11,51,59], including formation of active Cdc42-GTP patch through GEF-mediated nonlinear positive feedback, competition of different patches for a limiting component, and GAP-mediated negative feedback. This combination of feedbacks leads to patches of Cdc42-GTP that oscillate out of phase, as occurs transiently in budding yeast before the establishment of a dominant patch [51]. However, the overall dynamics we obtain are somewhat different and include the NETO polarity transition as well as stable symmetric and asymmetric oscillations. Another difference is the more prominent role of membrane diffusion of Cdc42-GDP and GAPs across the whole cell in our fission yeast model, as opposed to the diffusion in the cytoplasm and the patch region in budding yeast. How non-equilibrium fluxes through the cytoplasm versus the membrane impact pattern formation dynamics could be a topic for further investigation. Another similarity to prior budding yeast models, is the control of patch width through side GAP accumulation (implemented through different transport mechanisms): Budding yeast controls Cdc42 patch size in part through an inhibitory ring of septin-bound GAPs around the zone of Cdc42 activation [59].

A question of relevance in the broader context of cellular morphogenesis and its regulation by negative feedbacks, is why fission yeast uses GAPs with such different membrane localizations for its polarization process. In principle, one negative regulator could be sufficient for oscillations, for example models of the bacterial Min system, using similar reaction–diffusion mechanisms to this work, reproduce tip to tip MinD oscillations with MinE as a single negative inhibitor [27]. One negative inhibitor was also sufficient to contain and localize an activation zone in our prior model of Ras1 mating patch exploratory dynamics through fast diffusion of Gap1 around the active Ras1 zone [41]. Perhaps the combination of cell tip and cell side inhibitors allows for better control of a localized activation region with precise size over μm scales, as needed for stable tubular projections. Prior theoretical analysis further suggests that stability of cell diameter over successive divisions requires that the growth zone width do not vary strongly with cell diameter [60]. For example, Figure 7 shows how reduction of GAP_II_ recruitment rate can promote a wider Cdc42 patch, features seen in cells with deleted Rga4, which have a wider Cdc42 patch and cell diameter. To further illustrate the implications of GAP regulation in the model, consider the case where relaxing the assumption of tip-biased activation leads to a localized stable or oscillating patches of Cdc42-GTP surrounded by a ring of high concentration of GAP_II_ (Figure 8A). Further eliminating GAP_II_ by setting $k_{5}^{n}=0$ results in a more diffuse Cdc42-GTP zone that moves as a traveling wave around the cell surface, chased by the only remaining inhibitor GAP_I_ (Figure 8B). Thus, the system changes qualitative behavior, similar to the traveling Rho waves in larger cells [61] and to the reconstituted traveling Min waves [27], which can also be described by reaction diffusion equations [27,61]. We thus, speculate that use of multiple GAPs allows for a robust dynamical landscape that suits fission yeast’s tubular growth pattern, under conditions when this might not be possible by positive feedback and geometry alone [62,63].

## Figures and Tables

**Table 1:** List of parameters in reaction–diffusion equations, their reference value in the simulations, and their physical meaning in the model. Unless indicated, the values were estimated or adjusted to match experimental observations as mentioned in the main text.

Variable	Reference Value	Description
$D_{T}$	0.02 μm^2^/s	Diffusion coefficient of Cdc42-GTP from [33]
$D_{D}$	0.2 μm^2^/s	Diffusion coefficient of Cdc42-GDP from [33]
$D_{{\mathrm{GAP}}_{I}}$	0.03 μm^2^/s	Diffusion coefficient of GAP_I_, estimated
$D_{{\mathrm{GAP}}_{\mathrm{II}}}^{\mathrm{fast}}$	0.0625 μm^2^/s	Diffusion coefficient of fast GAP_II_, estimated
$D_{{\mathrm{GAP}}_{\mathrm{II}}}^{\mathrm{slow}}$	0.005 μm^2^/s	Diffusion coefficient of slow GAP_II_, estimated
$k_{1}^{n}$	0.000625/s	Spontaneous rate Cdc42-GTP hydrolysis, adjusted
$k_{2}^{n}$	0.00325 μm^2^/s	Rate constant of GAP_I_-mediated Cdc42-GTP hydrolysis, adjusted
$k_{3}^{n}$	0.00125 μm^2^/s	Rate constant of fast GAP_II_-mediated Cdc42-GTP hydrolysis, adjusted
$k_{4}^{n}$	250/s	Rate constant of Cdc42-GTP-mediated GAP_I_ recruitment, adjusted
$k_{5}^{n}$	0.03/s	Rate constant of Cdc42-GTP-mediated GAP_II_ recruitment, adjusted
$k_{6}^{n}$	2/s	Rate of fast GAP_II_ conversion to slow form, adjusted
$k_{7}^{n}$	0.025 μm^2^/s	Cdc42-GTP-mediated conversion of slow to fast GAP_II_, adjusted
$k_{8}^{n}$	0.0005 μm^2^/s	Rate constant of slow GAP_II_-mediated Cdc42-GTP hydrolysis, adjusted
$k_{\mathrm{sat}}$	600/μm^2^	Saturating concentration of GAP_I_ negative feedback, adjusted
$k_{0}^{p}$	0.0025 μm^2^/s	Rate constant of GEF-mediated Cdc42-GDP activations, adjusted
$k_{1}^{p}$	0.5 μm^3^	Linear rate constant of GEF recruitment to Cdc42-GTP, adjusted
$k_{2}^{p}$	0.1 μm^5^	Quadratic rate constant of GEF recruitment to Cdc42-GTP, adjusted
$E_{c}{}^{\mathrm{tot}}$	250	Total pool of GEFs, estimated
$j_{D}^{p}$	2.4/s/μm^2^	Flux of Cdc42-GDP from cytoplasm to membrane, estimated
$r_{T}$	0.005/s	Rate of Cdc42-GTP dissociation from membrane from [33]
$r_{D}$	0.03/s	Rate of Cdc42-GDP dissociation from membrane from [33]
$r_{{\mathrm{GAP}}_{I}}$	0.01/s	Rate of GAP_I_ dissociation from membrane, adjusted
$r_{{\mathrm{GAP}}_{\mathrm{II}}}^{\mathrm{fast}}$	0.0125/s	Rate of fast GAP_II_ dissociation from membrane, adjusted
$r_{{\mathrm{GAP}}_{\mathrm{II}}}^{\mathrm{slow}}$	0.0025/s	Rate of slow GAP_II_ dissociation from membrane, adjusted
$r_{\mathrm{noise}}$	0.0021/s	Rate of random Cdc42-GDP conversion to Cdc42-GTP, adjusted
λ	2.5 μm	GEF activation scale at cell tips, estimated
*L*	8 μm	Cell length

## Supplementary Materials

The following are available online at https://www.mdpi.com/2073-4409/9/8/1769/s1, Video S1: Simulation of Cdc42-GTP and total GAP_II_ corresponding to Figure 3A.
